# Feasibility of computerized clinical decision support for pediatric to adult care transitions for patients with special healthcare needs

**DOI:** 10.1093/jamiaopen/ooab088

**Published:** 2021-11-03

**Authors:** Nikolas J Koscielniak, Ajay Dharod, Adam Moses, Richa Bundy, Kirsten B Feiereisel, Laurie W Albertini, Deepak Palakshappa

**Affiliations:** 1 Clinical and Translational Science Institute, Wake Forest School of Medicine, Winston-Salem, North Carolina, USA; 2 Department of Internal Medicine, Wake Forest School of Medicine, Winston-Salem, North Carolina, USA; 3 Department of Implementation Science, Wake Forest School of Medicine, Winston-Salem, North Carolina, USA; 4 Center for Biomedical Informatics, Wake Forest School of Medicine, Winston-Salem, North Carolina, USA; 5 Center for Healthcare Innovation, Wake Forest School of Medicine, Winston-Salem, North Carolina, USA; 6 Department of Pediatrics, Wake Forest School of Medicine, Winston-Salem, North Carolina, USA; 7 Department of Epidemiology and Prevention, Division of Public Health Sciences, Wake Forest School of Medicine, Winston-Salem, North Carolina, USA

**Keywords:** clinical decision support, healthcare transition, learning health systems, pediatrics, electronic health records

## Abstract

The objective of this study was to determine the feasibility of a computerized clinical decision support (cCDS) tool to facilitate referral to adult healthcare services for children with special healthcare needs. A transition-specific cCDS was implemented as part of standard care in a general pediatrics clinic at a tertiary care academic medical center. The cCDS alerts providers to patients 17–26 years old with 1 or more of 15 diagnoses that may be candidates for referral to an internal medicine adult transition clinic (ATC). Provider responses to the cCDS and referral outcomes (e.g. scheduled and completed visits) were retrospectively analyzed using descriptive statistics. One hundred and fifty-two patients were seen during the 20-month observation period. Providers referred 87 patients to the ATC using cCDS and 77% of patients ≥18 years old scheduled a visit in the ATC. Transition-specific cCDS tools are feasible options to facilitate adult care transitions for children with special healthcare needs.

## INTRODUCTION

 Adolescents and young adults with special healthcare needs are increasingly surviving into adulthood and require increased support to effectively transition from pediatric to adult care.^1–5^ A healthcare transition is defined as the “planned, purposeful process in which adolescents and young adults move from pediatric-focused to adult-focused healthcare delivery”.^6–8^ However, deficiencies exist in the healthcare transition process, such as insufficient measurement of transition readiness, inadequate transition preparation, and time gaps between clinic visits.^7^^,^^9–11^ A learning health systems approach could assist with this process by harnessing key data and knowledge about children with special healthcare needs to improve the initiation and facilitation of healthcare transitions.^12–17^

A key component of any healthcare transition program is a well-planned and executed mechanism to track and measure readiness of adolescents throughout the process of transition and transfer.^2^ Unfortunately, literature demonstrates a lack of surveillance, structure, and support for these patients, which contributes to the development of new co-morbidities and adverse events.^10^^,^^18–24^ While 750 000 children transition into adulthood annually,^25^ support for pediatric to adult care transitions is relatively low,^8^^,^^26^ but care models to support transitions are increasing.^2^^,^^3^^,^^11^^,^^24^^,^^27–29^ Digital health tools and clinical decision support systems are advancing to support the delivery of care to patients with chronic diseases.^1^^,^^28^

Clinical decision support systems are integral to early childhood screening and automating many other decision-making processes in pediatric healthcare settings.^30–33^Although computerized clinical decision support (cCDS) has effectively improved many processes in pediatrics, the utilization and implementation of cCDS tools to support pediatric to adult care transitions are limited.^27^

To address this gap, we implemented a healthcare transitions cCDS tool in the electronic health record (EHR) in 1 academic general pediatrics clinic (GPC). This study was part of a larger clinical initiative to improve the care of patients with childhood-onset chronic conditions as they transition from pediatric to adult care. Transitioning from pediatric to adult care is a multi-factorial process, including patient, provider, system, and psychosocial factors.^4^^,^^21^ As an initial step, we developed the cCDS tool to identify patients at risk of developing worse outcomes if they are not successfully transitioned, facilitate the transfer of care to an adult provider, and have the ability to monitor the process. The cCDS triggers when a patient of transition age with one or more chronic disease diagnoses presents to the GPC and supports rapid decision-making around referral to a recently established adult transition clinic (ATC).

## MATERIALS AND METHODS

### Study design

We conducted a retrospective cohort study to determine the extent that the cCDS triggered to refer patients to an ATC that would serve as the patient’s adult primary care medical home. The cCDS was implemented as part of standard care at Wake Forest Baptist Medical Center and subsequently received Wake Forest University Institutional Review Board approval for retrospective analysis (IRB #00069131). The study period was from January 2018 through August 2020.

### Transitions cCDS procedure

**Figure 1.**
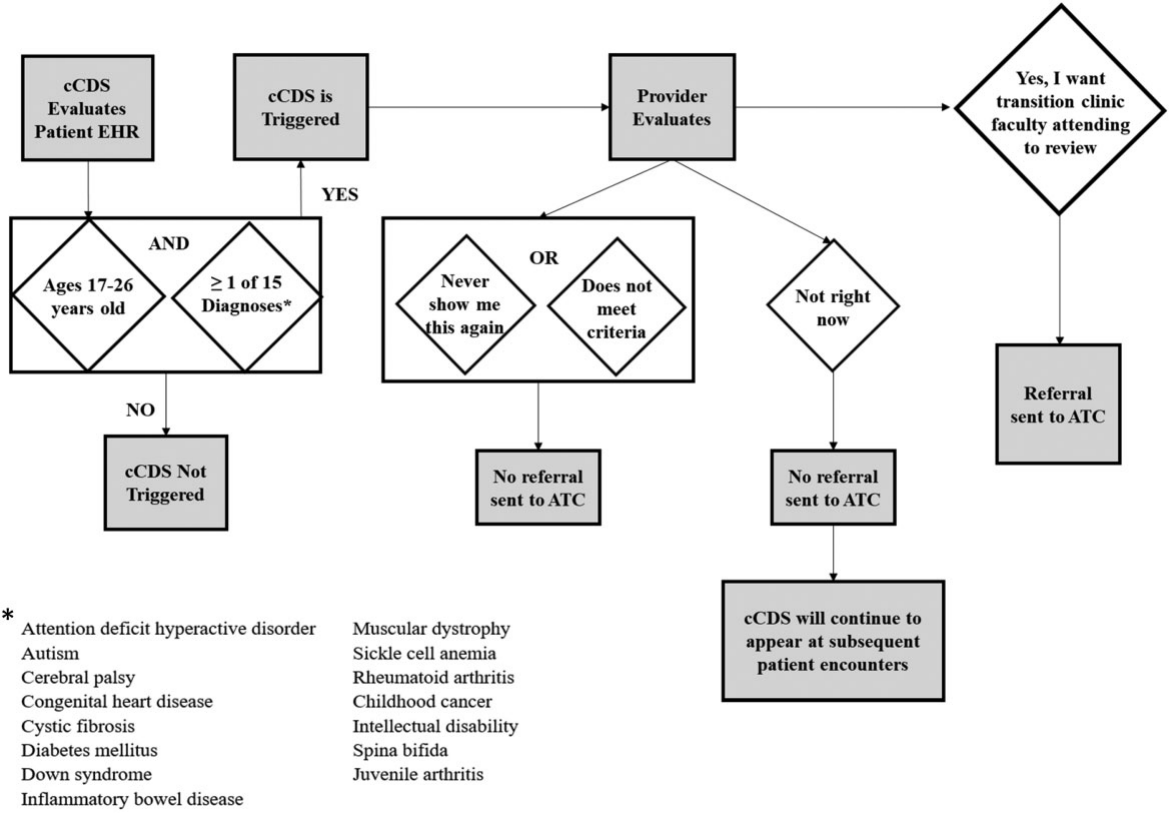
Flow diagram of the computerized clinical decision support tool for supporting referral to an adult transition clinic.

The cCDS alerted the provider when a patient was between 17 and 26 years old and had any 1 of 15 diagnoses (attention deficit disorder, autism, cerebral palsy, congenital heart disease, cystic fibrosis, diabetes, down syndrome, inflammatory bowel disease, muscular dystrophy, sickle cell anemia, rheumatoid arthritis, childhood cancer, intellectual disability, spina bifida, juvenile arthritis). These 15 diagnoses were selected after multiple meetings with stakeholders (including pediatricians, adult primary care providers, clinic administrators, and subspecialists) across the institution because: (1) they were common diagnoses seen in pediatrics but there were often challenges with transitioning them to an adult provider; (2) were diagnoses that adult primary care providers did not commonly manage; or (3) received subspecialty care at the institution but often lacked a primary care medical home. Diagnoses were defined as groups of diagnoses using standard ICD-10 and/or SNOMED codes. Upon opening a chart in the EHR, the cCDS alerts the pediatric provider to select 1 of 4 responses about whether the patient is a candidate for referral to the ATC: “Yes, I want [ATC Attending] to review”, “Never show me this again”, “Does not meet criteria”, and “Not at this time” (Figure  1). If the provider selects “Yes”, an EHR in-basket message is sent directly to the ATC attending, and no further cCDS will trigger for that patient. Eligible patients (who had 1 or more of the 15 diagnoses and were ≥17 years of age) for the ATC were then contacted by the clinic to schedule an appointment. Alerts are silenced for a given patient after a provider selects “Yes” or “Never show me this again,” but alerts continued to trigger for future visits when a provider selects “Not at this time” or “Does not meet criteria”.

EHR data were extracted for all visits in which the cCDS was triggered. We extracted patient’s gender, age, race, and health insurance status. In addition to GPC visit-related data and responses to the cCDS, we also extracted data on the scheduled and completed ATC visits.

Prior to the development of the ATC and the cCDS tool, all well-child documentation included a “SmartData” element for whether the provider discussed transition to adult care services. The “Transition Discussed” data element had 3 possible responses: “Yes—transition around age 18”, “Yes—transition plan discussed”, and “No”. We extracted data for this data element from the EHR to determine if referrals through the cCDS tool were associated with transition discussion documentation.

### Data analysis

We calculated descriptive statistics for all demographic characteristics at the last visit when the cCDS was triggered. Feasibility was determined by identifying the reach of the cCDS, or the proportion of visits and unique patients for which the cCDS was triggered. The final provider response to the cCDS for each patient was stratified to determine the proportion of patients that pediatric providers referred to the ATC. For referred patients, we identified the visit type (office visit, phone call, telehealth, etc.) at which the referral was made and calculated the frequency of patients who were scheduled and completed a visit to the ATC. We also determined inconsistencies in provider responses and referral to the ATC by flagging patients whose providers did not respond “Yes” but scheduled a visit and presented to the ATC. Lastly, for referred patients, we evaluated whether the cCDS influenced the documentation of and provider responses to the Transition discussed data element. All analyses were conducted using R 3.6.2 and R Studio statistical software.^34^

## RESULTS

The cCDS was triggered for 152 unique patients spanning 232 visits over the 20-month observation period. The majority was male, Black race, and received Medicaid insurance (Table  1). The average patient age was 17.6 years old (±1.4), and 104 (68.4%) patients were 17 years old at their last visit when the cCDS was triggered.

**Table 1.** Demographic characteristics and responses to transitions cCDS for the last visit the cCDS was triggered

	*N* (%)	Referred to ATC Via cCDS	Total Referred to ATC	Scheduled ATC Visit	Presented to ATC
Patients	152	87	91	34	19
Sex					
Male	81 (53.3)	43	47	19	10
Female	71 (46.7)	44	44	15	9
Race					
White	23 (15.1)	10	10	3	1
Black	66 (43.4)	36	38	18	10
Asian	1 (.06)	–	–	–	–
Other	62 (40.7)	41	43	13	8
^a^Visit age at last cCDS					
17	104 (68.4)	56	56	6	–
18	33 (21.7)	24	27	20	11
19	5 (3.3)	1	2	2	3
20	4 (2.6)	3	3	3	2
21–26	6 (3.9)	3	3	3	3
Insurance status					
Medicaid	116 (76.3)	72	75	25	12
Other	26 (17.1)	10	10	6	4
None	10 (6.6)	5	6	3	3
Transition cCDS response					
Yes, I want the ATC attending to review	82 (54.0)	87	87	30	17
Not at this time	50 (32.9)	–	4	4	2
Does not meet criteria	13 (8.6.)	–	–	–	–
Never show me this again	2 (1.3)	–	–	–	–

^a^ Columns for visit age relate to the patient age at last visit where the cCDS fired. Therefore, ages relate to the pediatric visit and not the time when the ATC visit was scheduled or completed.

ATC: adult transition clinic; cCDS: computerized clinical decision support.

Of the 152 patients, GPC providers responded that 13 (8.6%) were not transition candidates, 50 (32.9%) were not ready for transfer, and 2 (1.3%) did not meet criteria. These 65 patients remained in the pediatrics clinic during the study period. Eighty-seven patients (57.2%) were referred to the ATC through the cCDS, 55 (63.2%) were referred at the first instance the cCDS triggered, and 32 (36.8%) on or after the second instance.

Of the 87 patients referred, 30 patients (34.5%) scheduled a visit in the ATC, 19 (21.8%) of whom completed a visit. The cCDS was effective in referring 31 patients 18 years or older (and eligible to be scheduled in the ATC) and 24 (77%) of these patients scheduled a visit. Of the 57 patients who were referred but did not schedule a visit in the ATC, 38 (66.7%) continued to be seen in pediatrics, 1 (1.8%) was seen in another adult primary care practice, 1 (1.8%) in a family medicine practice, 5 (8.8%) in a subspecialty clinic, and 12 (21.1%) were lost to follow-up. At the end of the study period, 50 patients had not scheduled a visit, as of yet, in the ATC because they were still 17 years old. Four patients who were not referred through the cCDS were scheduled for a visit in the ATC, 2 of which completed a visit.

The transition discussed data element was populated in ∼19% of all GPC visits (data not presented). Providers populated the transition discussed data element in clinical notes for 22 of the 87 patients referred for adult care transition services through the cCDS. Of these 22 patients, providers indicated that 18 should transition around age 18, a transition plan was discussed for 1 patient, and transition was not discussed for 3 patients.

## DISCUSSION

In this pilot study, we demonstrated the feasibility of a cCDS for initiating adult care transitions for patients with special healthcare needs, as 77% of patients who were eligible and referred through the cCDS scheduled a visit. Although the cCDS supports identifying eligible patients and provider decision-making about the healthcare transition process, barriers remain to the full transfer of care from pediatric to adult services as only 20% of patients referred completed a visit. This is the first study that we know of which uses cCDS to support referral of pediatric patients with special healthcare needs to adult care services. Future research should continue to build a learning health system around healthcare transitions.

Our findings also show that clinical documentation practices may play a vital role in surveillance of care transition readiness and transfer processes. The sparse documentation of the transition discussed data element for those patients referred to ATC services through the transition cCDS demonstrates that the cCDS did not influence or improve provider documentation of the transition discussed data element. Reasons for why providers did not document this data element are complex and may stem from tensions in documentation of structured and unstructured data, limited awareness of available codable data concepts in the EHR interface, lack of feedback loops to inform providers of how these data are used, and low perceived value of documenting about transition preparation and care transfer. However, these hypotheses require further research. Additionally, with the recent policy changes from 21st Century Cures Act giving patients more access to provider clinical notes and decision-making about their care,^35^ improving patient transition readiness and documentation will be increasingly important in the future. The role of provider transparency and communication about a child’s readiness to transition to adult care services is magnified and creates an opportunity for parents and their children to become more active participants in the decision-making process on healthcare transitions.

The cCDS appears to be a more feasible option to capture provider responses about patient readiness to transition to adult care services, rather than documenting transition-related coded data concepts in the EHR. Previous studies using cCDS, such as the Child Health Improvement through Computer Automation (CHICA) tool, demonstrated that these decision tools can improve screening rates for a variety of pediatric conditions and even automate the identification and screening of pediatric patients at high risk for conditions like type 2 diabetes.^30^^,^^31^^,^^33^^,^^36^ Previous reports indicate that preparation for transition may start at 14 years old or younger,^2^ and it is expected that adolescents transition to adult services between 17 and 19 years old.^37^ Yet, the digital infrastructure to support pediatric providers in making decisions about transition preparation, readiness, and initiating a care transfer to adult services is insufficient. Expanding this digital infrastructure is imperative to realize a learning health system^16^ for healthcare transition, especially through innovating the use of cCDS to support the routine capture of critical data for measurement and tracking of care transition readiness and care transfers.

There are several limitations to this study that should be acknowledged. First, this study was conducted at 1 clinic, so may not be generalizable to other clinics. Second, we did not include a control group and cannot account for temporal trends. Third, we only analyzed structured clinical data that providers populated via the cCDS and discrete data elements included in the well-child templates. We did not evaluate the narrative clinical notes for content on transitions. Analyzing narrative clinical notes may reveal important findings about transition-related delivery processes, how pediatric and adult providers communicate readiness to transition, and the extent that the cCDS influences providers to discuss and document information on patient transition. A qualitative analysis of the clinical documentation of healthcare transitions beyond structured data fields for this population is currently underway. Further research is needed to modify the tool to support patients in fully transitioning to adult care and to understand how clinical decision tools could be used to address barriers to transitions.

Our findings indicate that informatics tools such as cCDS may be beneficial for healthcare transition and transfer research and practice because they can support improved infrastructure for patients at a vulnerable time in their lives. Future work should focus on integrating transition readiness instruments into cCDS form for screening, tracking, and facilitating transition and care transfers.

## FUNDING

The National Center for Advancing Translational Sciences of the National Institutes of Health under Award Number 5TL1TR003136 (to NJK). The National Heart, Lung, and Blood Institute of the National Institutes of Health under Award Number K23HL146902 (to DP). The content is solely the responsibility of the authors and does not necessarily represent the official views of the National Institutes of Health. The funding organization has no role in the design and conduct of the study; collection, management, analysis, and interpretation of the data; preparation, review, or approval of the manuscript; and decision to submit the manuscript for publication. Ms. Amy Ayler with the Ambulatory Information Technology Services team helped create the cCDS.

## AUTHOR CONTRIBUTIONS

All authors have contributed to this manuscript and approved the version of this submission. NJK contributed to the conception and design of the study, conducted the data analysis, interpretation of the data, and drafted the initial version of the manuscript. AM and RB contributed to data collection, interpretation of the data, and critical revision of the manuscript. AD designed, created, and implemented the cCDS with assistance from Amy Ayler. AD, KBF, LWA, and DP contributed to the conception and design of the study, interpretation of the data, and critical revision of the manuscript.

## CONFLICT OF INTEREST STATEMENT

None declared.

## DATA AVAILABILITY

The data underlying this article cannot be shared publicly due to the nature of this research and because participants of this study did not agree for their data to be shared publicly. Additionally, data sharing is not applicable because no new data were created in this study.

## Notes

Dr. Nikolas Koscielniak, PhD, MPH, is a program officer at the Patient‐Centered Outcomes Research Institute (PCORI). The work described in this manuscript was completed while Dr. Koscielniak was a Postdoctoral Fellow at Wake Forest School of Medicine and does not necessarily represent the views of PCORI, its Board of Governors, or Methodology Committee.
